# The Utilization of Topical Insulin for Ocular Surface Diseases: A Narrative Review

**DOI:** 10.7759/cureus.62065

**Published:** 2024-06-10

**Authors:** Kayvon A Moin, Srujay Pandiri, Garrett N Manion, Alex H Brown, Majid Moshirfar, Phillip C Hoopes

**Affiliations:** 1 Hoopes Vision Research Center, Hoopes Vision, Draper, USA; 2 Ophthalmology, University of Missouri Kansas City School of Medicine, Kansas City, USA; 3 Ophthalmology, Creighton University School of Medicine, Omaha, USA; 4 Ophthalmology, University of Arizona College of Medicine - Phoenix, Phoenix, USA; 5 Eye Banking and Corneal Transplantation, Utah Lions Eye Bank, Murray, USA; 6 Corneal and Refractive Surgery, Hoopes Vision Research Center, Draper, USA

**Keywords:** substance p, insulin-like growth factor, corneal epithelial defect, topical insulin, corneal wound healing, limbal epithelial stem cell, persistent epithelial defect, neurotrophic keratopathy, dry eye disease, diabetic keratopathy

## Abstract

Various etiologies, including diabetic keratopathy (DK), dry eye disease (DED), and neurotrophic keratopathy (NK), can disrupt corneal homeostasis, exacerbating corneal epithelial defects. Topical insulin has emerged as a promising therapy for promoting corneal wound healing and addressing underlying pathologies. This review systematically evaluates the efficacy of topical insulin across different corneal disorders. A literature review was conducted across the PubMed, Google Scholar, and Scopus research databases. The search resulted in a total of 19 articles, consisting of clinical trials, retrospective studies, and case reports. In DK, topical insulin accelerates corneal wound healing post-vitreoretinal surgery with lower concentrations showing higher outcomes when compared to conventional therapy, possibly due to improved epithelial stem cell migration. In comparison, the dry-eye disease results are inconclusive regarding patient-reported outcomes and corneal staining. For NK, topical insulin accelerates corneal wound healing and restores corneal nerve sensation. Other persistent epithelial defect (PED) etiologies that have been treated with topical insulin are infection, immune-mediated, mechanical and chemical trauma, and chronic ocular surface alterations. Although individual mechanisms for the benefits of topical insulin for each of these etiologies have not been studied, the literature demonstrates that topical insulin is efficacious for PEDs regardless of etiology. Future clinical trials need to be conducted to further evaluate optimal dosing, duration, and use of topical insulin for the restoration of the corneal surface.

## Introduction and background

The translucency and refractive power of the human cornea is essential for vision [1]. The cornea is avascular and maintains its refractive surface by constantly regenerating its five- to seven-layer stratified nonkeratinizing squamous epithelium, in which the average cell is replenished every seven to 10 days [2]. The regenerative process is enabled by the complex cellular composition within the base of the limbus, where limbal epithelial stem cells (LESCs) signal with mesenchymal stromal cells, T cells, Schwann cells, and nerves, among others [3]. Within this limbal niche, LESCs can rapidly differentiate into transient amplifying cells upon injury-based signaling which migrate centripetally to replenish the basal cells of the corneal epithelium [4,5]. LESCs start to proliferate, differentiate, and migrate to the site of injury shortly after corneal damage occurs. The specific rate of migration can vary based on the extent of the injury and the individual's healing response. In a study where the migration of labeled LESCs was tracked, it was observed that these cells moved centripetally at a rate of about 0.73±0.1 mm per day [6].

Corneal epithelial injuries can occur from numerous etiologies, from physical or chemical injury to underlying pathologies. A persistent epithelial defect (PED) happens when there is a failure of re-epithelialization and closure past a normal timeframe, around 10-14 days. This condition results in an area of the cornea where the epithelial layer remains continuously open or unhealed despite treatment [7]. This can be quite problematic, as PEDs can lead to myofibroblast proliferation and disordered extracellular matrix deposition, resulting in scarring, loss of vision, and increased risk of infection or perforation [8,9]. Development of PEDs can be in part due to the failure of corneal protective mechanisms, such as tear film integrity, corneal innervation, the epithelial regenerative system, or balance of molecular factors like cytokines, enzymes, and adhesion molecules [10]. PEDs have traditionally been treated with aggressive lubrication and bandage contact lenses [7].

Conditions that affect tear film integrity include dry eye disease (DED), which systemic conditions like Sjogren’s syndrome can contribute to by causing lacrimal dysfunction. DED is largely an issue of tear homeostasis, resulting in excess evaporation or insufficient production of tears [11]. Both hyperosmolarity, which can trigger inflammatory signaling, and lack of lubrication, which can result in excess friction during blinking, may damage the epithelium [12]. This damage leads to discomfort often described as grittiness or burning [13]. DED prevalence has been reported to be around 5%, with a female predominance [14]. Treatment for DED includes a combination of traditional therapies like artificial tears and punctal occlusion, anti-inflammatory and immunomodulatory agents, as well as emerging strategies such as hormonal therapy, neuromodulation, stem cell treatments, and various surgical techniques [15-18].

In addition to sensation, corneal nerves are important for maintaining corneal integrity, as they activate reflex tearing, protective blinking, and trophic factors that regulate the activity of LESCs [19,20]. Disruption of corneal innervation by injury, systemic disease, infection, and neurogenesis can lead to neurotrophic keratopathy (NK). In NK, the balance of epithelial trophic factors and mediators between LESCs and branches of the trigeminal nerve that enable re-epithelialization of the cornea is disrupted and can result in PEDs [21]. NK is rare and difficult to treat, but many new treatments are being explored. Depending on the stage of the disease, the various treatment options include medical therapies such as nerve growth factor drops, autologous serum drops, Substance P, insulin-like growth factor-1, matrix regenerating agent drops, and surgical procedures like amniotic membrane transplantation and corneal neurotization [21].

Another disease that can alter the cornea’s protective system is diabetes mellitus. While diabetic retinopathy affects over 20% of diabetics, the most common ocular manifestation of diabetes is diabetic keratopathy (DK), affecting 40%-70% of diabetics [22,23]. Its effects can widely range in severity, from superficial punctate keratitis to PEDs. The systemic hyperglycemic state present in diabetes affects the cornea in multiple ways. The hyperglycemia-induced mitochondrial overproduction of superoxide and the presence of advanced glycation end products cause inflammation and microvascular damage [24]. This alters the immune response and protein expression of corneal epithelial cells and may damage nerves [25]. The presence of peripheral neuropathy in diabetes is a strong predictor of DK [26]. Ultimately, hyperglycemic damage impairs corneal epithelial regeneration and sensation, which may heavily contribute to corneal pathologies [23]. As DK can affect the integrity of corneal innervation, it can contribute to NK as well. Traditional treatment of DK includes lubrication, reducing exposure, and tight glycemic control. Newer treatments currently under investigation include prophylactic antibiotics, stem cells, autologous cells, and topical treatments containing growth factors, cytokines, insulin, naltrexone, and nicergoline [27].

There is a wide range of etiologies of PEDs in addition to those previously mentioned. When considering a broad differential, many types of conditions may contribute to PEDs. These include 1) mechanical trauma, such as from trichiasis or foreign bodies, 2) iatrogenic causes, such as topical antibiotics, 3) excessive exposure, such as from nerve palsies causing incomplete lid closure, proptosis, and lagophthalmos, 4) limbal stem cells deficiency, such as from surgical complications or chronic contact lens use, 5) inflammatory conditions, such as Stevens-Johnson syndrome (SJS) or rheumatoid arthritis, 6) occupational hazards, such as light from welding, and 7) infections, such as latent viruses or resistant bacteria, though this list is not exhaustive [7].

Addressing the underlying cause of a PED can greatly assist healing. Novel treatments for many PED etiologies are now being developed, including those described for NK and DK. Topical insulin is one of these novel treatments currently being explored for use in many etiologies of PEDs. For example, the use of topical insulin is now being utilized after corneal epithelial debridement in retinal surgery, as postoperative recovery is often difficult in patients with diabetes [28].

**Figure 1:**
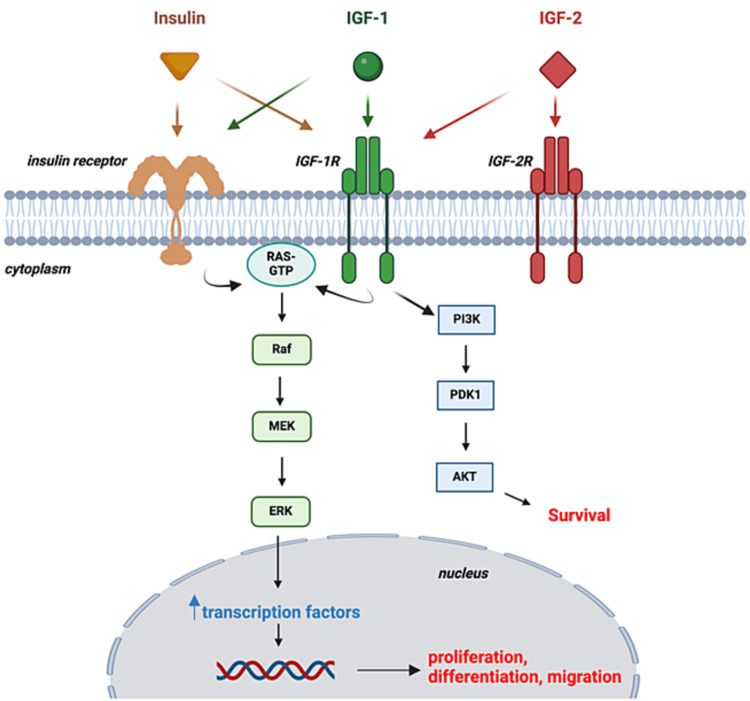
Topical insulin biochemical mechanisms for corneal wound healing Insulin, IGF-1, and IGF-2 can cross-bind to their respective receptors and initiate certain biochemical pathways inside the corneal epithelial cytoplasm. The RAS/ERK pathway promotes corneal epithelial cell proliferation, differentiation, and migration, whereas the PI3K pathway promotes anti-apoptosis and cell survival. IGF-1 = insulin-like growth factor 1; IGF-1R = insulin-like growth factor 1 receptor; IGF-2 = insulin-like growth factor 2; IGF-2R = insulin-like growth factor 2 receptor. Created with BioRender.com by the authors.

The corneal epithelium possesses neurotrophic factors expressed through a complex web of metabolic pathways that activate corneal healing processes [29]. Insulin-like growth factor (IGF)-1 and IGF-2, along with insulin, may potentially activate key pathways, such as the RAS/ERK and PI3K pathways, that promote corneal epithelial cell proliferation, differentiation, migration, and anti-apoptosis (Figure 1) [21]. The topical application of insulin leverages these metabolic pathways to promote corneal healing. Topical insulin has been shown to be superior to autologous serum tears and safe in dosages up to 100 IU/mL of short-acting insulin without any evidence of systemic absorption or hypoglycemia [30,31]. In general, lower insulin doses have been favored in many studies due to the lack of efficacy of high doses compared to lower ones, and recently 0.5-1 IU/mL has been recommended [28,30,32]. Studies have endorsed a significant re-epithelialization impact of topical insulin in PEDs, postoperative trauma, DK, NK, and other pathologies [32-36]. This review aims to provide a comprehensive review of indications, dosages, and formulations for the clinical use of topical insulin.

## Review

Methods

**Figure 2:**
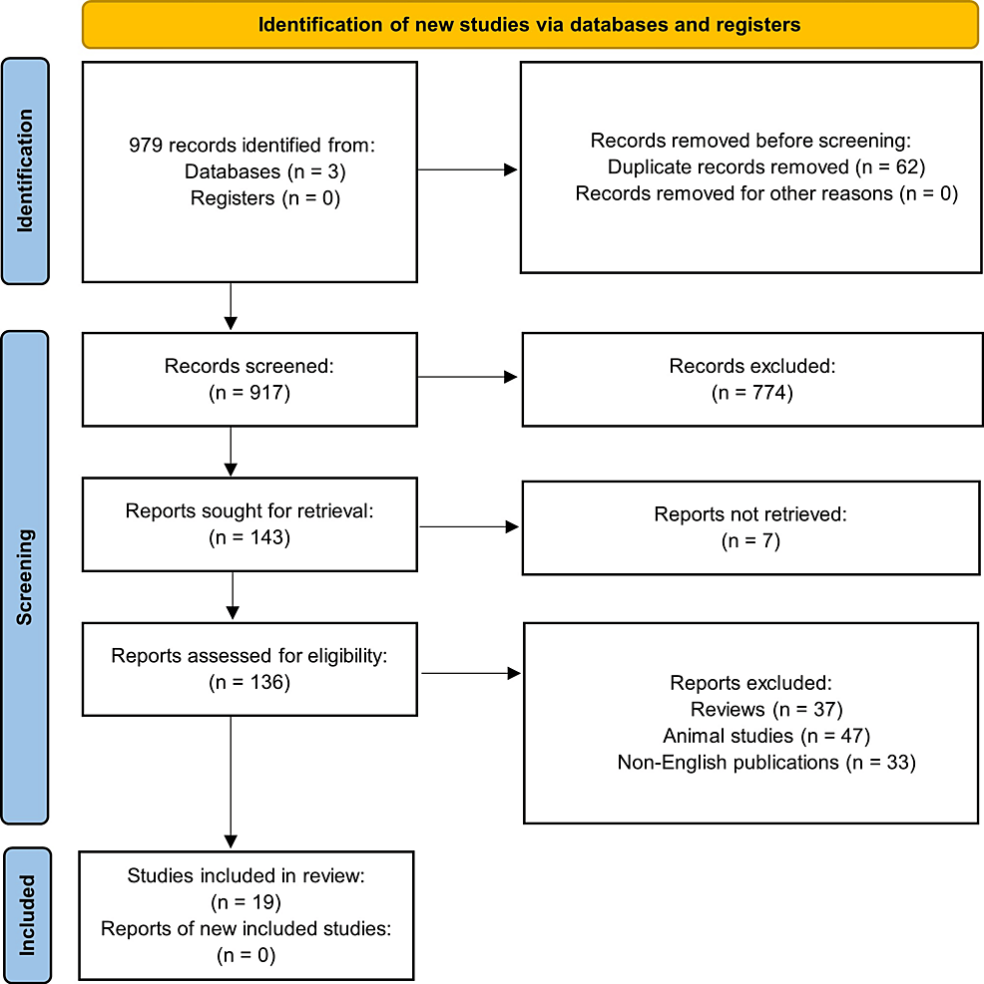
PRISMA flowchart process of literature search methodology Produced from prisma-statement.org by the authors.

This study was a systematic review of all currently published case reports, case series, clinical studies, and clinical trials, regarding the use of topical insulin in corneal diseases. We systematically searched PubMed, Google Scholar, and Scopus research databases, last accessed on April 18, 2024. The following search phrases were used: (topical insulin OR ophthalmic insulin OR insulin eye drop* OR insulin drop*) AND (cornea OR corneal OR ocular surface) AND (keratopathy OR keratitis OR diabetic keratopathy OR neurotrophic keratopathy OR infectious keratitis OR dry eye OR dry eye disease OR dry eye syndrome OR epithelial defects OR persistent epithelial defect* OR dystrophy OR corneal dystrophy). The search yielded a total of 979 total articles (148 PubMed, 733 Google Scholar, and 98 Scopus). Sixty-two duplicate articles, 774 irrelevant articles, seven articles with unretrieved PDFs, 37 review articles, 47 animal studies, and 33 non-English articles were excluded (Figure 2). One hundred thirty-six articles remained, and further screening for eligibility and relevance led to a final total of 19 articles. Any questions on study exclusions among the manuscript review team were resolved by the senior author (KM). Data extraction was performed by one author (SP) and was reviewed by the senior author (KM).

Results

*Diabetic Keratopathy (DK)* 

The literature search found a total of 121 eyes (120 patients) across four studies exploring the efficacy of topical insulin in healing corneal epithelial defects in diabetic patients after corneal debridement during vitreoretinal surgery (Table 1) [28,30,34,37]. The mean age of the patients studied was 57.12 ± 0.09 years (range: 31-75 years). One of these studies was a retrospective study of 14 patients (15 eyes) by Bastion and Ling, comparing the use of topical insulin 1 IU/mL four times daily in the diabetic study group (DTI) to conventional postoperative therapy (Dexamethasone 0.1% and ciprofloxacin HCl 0.3%) in the diabetic (DCT) and non-diabetic (NDCT) control groups [28]. Three of these studies were randomized control trials (RCTs), with sample sizes ranging between 32 and 38 patients, comparing topical insulin eye drops used four times daily to control groups using 0.9% normal saline, 0.15% sodium hyaluronate, Vismed (0.18% sodium hyaluronate) artificial tears for various durations of time (72-168 hours) [30,34,37]. Fai et al. compared three different concentrations (0.5, 1, 2 IU/mL) of topical insulin [34], whereas the other two RCTs by Quiroz-Mendoza et al. and Dasrilsyah et al. used only 0.5 IU/mL for their patients. All four studies utilized a recombinant human formulation of topical insulin [30,37]. The retrospective study focused on epithelial defect size as a primary outcome, whereas the RCTs focused on the time or rate of corneal epithelial wound healing. 

**Table 1:** Literature results for diabetic keratopathy and dry-eye disease For all results, the data have been represented as mean ± standard deviation QID = four times daily; RCT = randomized controlled trial; CS = case series

Author	Study Design	Patients (eyes)	Insulin Formulation/Dosage	Control Group	Primary Outcome	Results	Duration
Diabetic Keratopathy (DK)							
Bastion & Ling [28]	Retrospective study	14 (15)	Actrapid 1 IU/mL QID	Dexamethasone 0.1% and ciprofloxacin HCl 0.3%	Epithelial defect size (mm^2^)	24 hrs: 23.28 ± 9.96 vs. 47.72 ± 12.65; p = 0.009 60 hrs: 0 vs. 10.56 ± 10.10; p = 0.005	60 hr
Dasrilysah et al. [30]	RCT	38 (38)	Actrapid 0.5 IU/mL QID	0.18% Sodium hyaluronate artificial tears	Rate of wound healing (mm^2^/hr)	1.20 ± 0.29 vs. 0.78 ± 0.20; p < 0.001	
Fai et al. [34]	RCT	32 (32)	Actrapid 0.5, 1, 2 IU/mL QID	0.9% Normal saline (NS)	Rate of wound healing (mm^2^/hr)	0.5 IU vs. NS: 1.80 ± 0.33 vs. 1.34 ± 0.44; p = 0.036	144 hr
Quiroz-Mendoza et al. [37]	RCT	36 (36)	Humulin R 0.5 IU/mL QID	0.15% Sodium hyaluronate artificial tears	Time until complete re-epithelialization (days)	3.0 ± 0.85 vs. 4.25 ± 0.62; p < 0.001	4 days
Dry-Eye Disease (DED)							
Azmi & Bastion [38]	RCT	160 (320)	Actrapid 1 IU/mL QID	Polyethylene glycol 400 4%/propylene glycol 0.3% artificial tears	Ocular Surface Disease Index (OSDI)	18.8 vs. 20.63; p = 0.453	4 weeks
Burgos-Blasco et al. [39]	CS	16 (32)	Regular Insulin 1 IU/mL QID	-	5-point rating of symptoms	3.4 ± 1.3 vs. 2.3 ± 1.0; p < 0.001	3 months

All studies yielded significant findings with their results. Bastion and Ling found a significant difference in epithelial defect size between the study group and DCT group at 24 (p = 0.009) and 60 hours (p = 0.005) [28]. The three RCTs found that topical insulin had significantly faster rates of epithelial wound closure when compared to the various formulations of artificial tears studied (p < 0.05) [30,34,37]. Interestingly, Fai et al. demonstrated through their comparisons of different topical insulin concentrations that the 0.5 IU/mL dose had the fastest rate of epithelial wound closure, whereas the 2 IU/mL dose had the slowest rate [34].

Dry Eye Disease (DED)

The literature search found a total of 352 eyes (176 patients) across two studies evaluating the use of topical insulin for the management of DED (Table 1) [38,39]. The mean age of the patients studied was 49.11 ± 0.47 years (range: 18-72 years). Azmi and Bastion conducted an RCT with 160 patients (320 eyes) over a duration of four weeks comparing Actrapid Insulin 1 IU/mL four times daily into the control group’s Polyethylene Glycol 0.4% and Propylene Glycol 0.3% artificial tear drops, with the Ocular Surface Disease Index (OSDI) score as the primary outcome [38]. Burgos-Blasco et al. looked at a case series (CS) of 16 patients (32 eyes) using regular insulin 1 IU/mL four times daily and evaluated patient-reported outcomes (PROs) and corneal staining findings at one and three months [39]. 

Azmi and Bastion found no statistical difference in OSDI scores between the topical insulin group and the controlled artificial tear group (p = 0.453) [38], whereas Burgos-Blasco et al. found that PROs and corneal staining findings had significantly improved after three months with topical insulin use (p < 0.001) [39]. However, it is important to note that 12 patients in the CS by Burgos-Blasco et al. were concurrently using autologous serum (AS), and 10 patients were using cyclosporine.

Neurotrophic Keratopathy (NK)

The literature search yielded 33 eyes (32 patients) across seven studies evaluating the use of topical insulin for the management of corneal ulcers or epithelial defects secondary to NK (Table 2) [33,35,40-44]. The mean age of patients studied was 53.64 ± 0.50 years (range: 2-73 years). NK in 76% of eyes (25 eyes) was due to herpes simplex virus (HSV), 15% (five eyes) was due to trigeminal nerve damage, 6% (two eyes) was due to diabetes, and 3% (one eye) was due to lagophthalmos. Five of these studies were case reports, in which various insulin formulations (regular, NPH, fast-acting) were used mainly at 1 IU/mL four to six times daily [33,40-43]. The duration of use ranged from one week to four months. It is important to note that Tong et al. used an unnamed formulation of insulin at a dosage of 25 IU/mL six times daily for their patient and Giannaccare et al. utilized a Hyper-CL soft contact lens concurrently with topical insulin use [40,43]. The last two studies were CS by Wang et al. and Soares et al., one with a sample size of six patients (six eyes) and the other 21 patients (21 eyes), that evaluated the use of insulin 1 IU/mL two to four times daily for refractory corneal ulcers for a duration of 25 and 45 days, respectively [35,44]. Soares et al. also evaluated time until complete re-epithelialization for NK stage 2 and NK stage 3 groups. All studies determined their results based on degree of re-epithelialization. 

**Table 2:** Literature search results for neurotrophic keratopathy and other etiologies For all results, the data have been represented as mean ± standard deviation CR = case report; HSV = herpes simplex virus; QID = four times daily; CS = case series; SJS = Stevens-Johnson syndrome; MMP = mucous membrane pemphigoid; AK = acanthamoeba keratitis; NRCT = non-randomized controlled trial

Author	Study Design	Patients (eyes)	Etiology	Topical Insulin Formulation/Dosage	Control Group	Primary Outcome	Results	Duration
Neurotrophic keratopathy (NK)								
Moreker et al. [33]	CR	1 (1)	Iatrogenic trigeminal nerve damage	Insulin aspart 1 IU/mL QID	-	Resolution of ulcer	Partial resolution	25 days
Soares et al. [35]	CS	21 (21)	HSV	Regular insulin 1 IU/mL QID	-	Time to resolution of defect/ulcer (days)	NK stage 2 vs. stage 3: 18 ± 9 vs. 29 ± 11; p = 0.025	7 – 45 days
Tong et al. [40]	CR	1 (2)	diabetes	Unspecified insulin 25 IU/mL 6x/day	-	Resolution of ulcer	Complete resolution	1 week
Khilji et al. [41]	CR	1 (1)	HSV	Regular insulin 1 IU/mL 6x/day	-	Resolution of ulcer	Complete resolution	2 months
Qasem et al. [42]	CR	1 (1)	HSV	Regular insulin 1 IU/mL 4-5x/day	-	Resolution of ulcer	Complete resolution	6 weeks
Giannaccare et al. [43]	CR	1 (1)	Iatrogenic trigeminal nerve damage	Humalog 1 IU/mL QID	-	Resolution of defect	Complete resolution	20 days
Wang et al. [44]	CS	6 (6)	HSV (2 eyes) trigeminal nerve damage (3 eyes) Lagophthalmos (1 patient)	Regular insulin 1 IU/mL 2-3x/day	-	Resolution of ulcer	Complete resolution	7 – 25 days
Other etiologies								
Diaz-Valle et al. [31]	Retrospective study	84 (?)	Infectious (26 eyes), neurotrophic (30 eyes), chronic alterations of ocular surface (24 eyes), immune mediated (5 eyes)	Fast-acting insulin 1 IU/mL 4x/day (51 patients)	Autologous serum (23 patients)	Time to resolution of epithelial defect (days)	32.6 ± 28.3 days vs. 82.6 ± 82.4 days; p = 0.011	13-231 days
Balal et al. [32]	CS	10 (11)	Chemical (5 eyes), SJS (2 eyes), MMP (3 eyes), AK (1 eye)	Regular recombinant insulin 1 IU/mL 4x/day	-	Time to resolution of epithelial defect (days)	62.3 ± 34.6 days for 9/11 eyes (82%); 1/11 had >60% reduction; 1/11 had no effect	21-210 days
Diaz-Valle et al. [45]	CS	21 (21)	Infectious keratitis (7 eyes), calcium keratopathy (5 eyes), ocular surgery (3 eyes), lagophthalmos (3 eyes), bullous keratopathy (2 eyes), herpetic eye disease (1 eye)	Regular insulin 1 IU/mL 4x/day	-	Time to resolution of epithelial defect (days)	34.8 ± 29.9 days for 17/21 patients (81%); 4/21 patients (19%) had partial closure, with mean 91.5% reduction	7-114 days
Esmail et al. [46]	NRCT	29 (58)	Recurrent epithelial erosions secondary to mechanical trauma (58 eyes)	Regular Insulin 1 IU/mL 3x/day	Cornetears gel 4x/day, gatifloxacin 5mg/dL 4x/day	Epithelial defect size (mm^2^)	Week 2: 0.64 ±1.0 vs. 2.36 ± 1.86; p = 0.006 Month 2: 0 vs. 1.33 ± 1.15; p = 0.046 Month 3: 0 vs. 3.0 ± 0; p = 0.002	3 months
Serrano-Gimenez et al. [47]	CR	1 (1)	Chemical injury (car battery spillage)	Regular Insulin 1 IU/mL 4x/day	-	Resolution of ulcer	Complete resolution	3 months

All studies displayed promising results with topical insulin use, with almost all patients achieving complete re-epithelialization of their corneal ulcers/epithelial defects with restoration of corneal sensation. Soares et al. reported that only 90% of their patients achieved complete re-epithelialization after 45 days of use [35]. However, they did report that the mean number of days until complete re-epithelialization was lower in the NK stage 2 group when compared with NK stage 3 (p = 0.025).

Other Etiologies 

A total of 175 eyes (145 patients) across five studies were found through the literature search describing topical insulin use for PEDs secondary to other etiologies (Table 2) [31,32,45-47]. 34.9% (61 eyes) were due to mechanical trauma, 19.4% (34 eyes) were due to an infectious etiology (bacterial, mycotic, acanthamoeba), 19.4% (34 eyes) were due to NK (herpetic, trigeminal nerve damage, ocular surgery, etc.), 17.7% (31 eyes) were due to chronic alterations to the ocular surface (i.e., calcium keratopathy, bullous keratopathy, drug toxicity, etc.), 5.1% (nine eyes) were immune-mediated (SJS, mucous membrane pemphigoid (MMP), etc.), and 3.4% (6 eyes) were due to chemical injury. The mean age of patients studied was 60.98 ± 0.39 years (range: 10-95 years). Two of these studies were CS: Diaz-Valle et al. observed 21 patients (21 eyes) who received regular insulin 1 IU/mL four times daily for two months, whereas Balal et al. observed 10 patients (11 eyes) who received recombinant human insulin 1 IU/mL four times daily for three months [32,45]. Diaz-Valle et al. assessed rate of PED closure and epithelial healing time as their primary outcomes, whereas Balal et al. evaluated resolution or improvement in PED. One of the other studies that was found was a retrospective study by Diaz-Valle et al. that treated 61 patients with Actrapid insulin 1 IU/mL and 23 control patients with AS and evaluated percent of patients in which epithelization was achieved, rate and time until epithelization, and need for amniotic membrane transplantation (AMT) in each group [31]. Esmail et al. conducted a non-randomized clinical trial that treated 29 patients (58 eyes) with regular insulin 1 IU/mL four times daily and a control group with Cornetears gel and evaluated resolution, healing time, and recurrence of PED at various follow-up time periods [46]. Lastly, the search found a case report by Serrano-Gimenez et al. in which regular topical insulin 50 IU/mL four times daily was used for PED secondary to a car battery spillage [47]. 

All studies found significant results with topical insulin use. Both case series showed that over 80% of their patients had complete closure of their PED defect, with Diaz-Valle et al. demonstrating a significant mean reduction in PED size between each week of evaluation (p < 0.05) [45]. The retrospective study by Diaz-Valle et al. showed that topical insulin was significantly superior to AS in regards to complete resolution (p = 0.002), time to resolution (p = 0.011), and need for AMT (p = 0.005) [31]. Esmail et al. demonstrated that topical was significantly superior to Cornetears and antibiotic eye drops at two weeks (p = 0.006), two months (p = 0.046), and three months (p = 0.002) [46]. The patient described in the report by Serrano-Gimenez et al. also achieved complete resolution of the PED after three months of topical insulin use [47].

Discussion

The area of study in which the applicability of topical insulin was studied the most was for PEDs secondary to DK. The studies demonstrated that topical insulin was superior to conventional postoperative therapy and various artificial tear formulations in diabetic patients with PEDs after vitreoretinal surgery in regard to complete and faster corneal wound healing. These findings are supported by an animal study by Zagon et al. which showed that diabetic rats achieved faster rates of wound healing with topical insulin compared to the untreated diabetic group [36]. The authors proposed that achieving normoglycemia in limbal stem cells restored DNA synthesis levels, allowing for the epithelial defect to accelerate the healing process. The association between hyperglycemia and LESC dysfunction has been thoroughly studied, showing that high glucose levels in these cells lead to a decrease in several LESC markers such as β1 integrin, neural cadherin and keratin [48]. As mentioned earlier, Fai et al. demonstrated that the 0.5 IU/mL dose of topical insulin had faster rates of epithelial healing compared to the 1 and 2 IU/mL doses [34]. One reason for this observation could be that the viscosity of higher concentrations slowed the migration of the epithelial stem cells during the wound-healing process [34]. Nonetheless, all groups achieved complete closure of their epithelial defects by the end of the study. Based on the clinical and animal studies described, it is evident that topical insulin eye drops have a clinical benefit for DK patients with PEDs. 

The findings for topical insulin use for DED were conflicting. Although Burgos-Blasco et al. showed significant improvements in PROs and corneal staining outcomes, their findings were confounded by concurrent AS and cyclosporine use in most of their patients [39]. Additionally, Azmi and Bastion displayed through their large sample that topical insulin had no additional benefits than their artificial tear formulations for DED, when evaluating OSDI scores [38]. Interestingly, an animal study by Cruz-Cazarim et al. showed that topical insulin 1 IU/mL/day restored tear fluid volume in diabetic rats with DED [49]. Other studies have concurrently shown that tear film volume, goblet cell number, and corneal glycocalyx area decrease secondary to diabetes-induced hyperglycemia [50]. These findings were supported by the discovery of insulin and IGF-1 receptors in the plasma membrane and cytoplasm of corneal and conjunctival epithelial cells by Rocha et al. [51]. The patients in the two studies that were found assessing topical insulin use for DED were not diabetic, thus questioning if topical insulin is truly efficacious in non-diabetic patients with DED. It seems that this medication may provide some degree of ocular restoration in patients who experience corneal dryness secondary to diabetes. Further clinical trials need to be performed to assess the efficacy of topical insulin for DED secondary to different inciting factors.

None of the studies that were found evaluating topical insulin use for PEDs secondary to NK utilized control groups. Nonetheless, most patients achieved reepithelialization of their epithelial defects and regained corneal sensation after topical insulin use. Zagon et al. demonstrated through their animal study that diabetic rats regained corneal sensation after topical insulin administration [36]. Restoration of corneal sensation was further demonstrated by Chen et al. who showed that daily topical insulin applied daily to the eyes of diabetic rats and mice prevented the depletion of nerves of the sub-basal plexus [52]. In vitro studies have shown that insulin binding to IGF-1 receptors in the cornea increases β-catenin in the epithelial cell cytoplasm, activating the Wnt signaling pathway, and subsequently increasing transcription factors such as c-Myc, cyclin D1, and Tcf4 to promote regeneration and repair of corneal nerve cells [53,54]. It is evident that topical insulin provides benefits for patients with PEDs secondary to NK. However, future clinical trials need to be conducted to evaluate the efficacy of topical insulin for PED secondary to NK as a first-line treatment. 

All studies that assessed PEDs secondary to other various etiologies utilized patients who were initially unresponsive to standard medical and surgical PED treatments. The most common etiology that was studied was mechanical trauma (34.9%), followed by infectious keratitis and NK (19.4% each). It is important to note that the two studies conducted by Diaz-Valle et al. included patients with PEDs secondary to NK [31,45]. Since their analyses were generalized for the PED group as a whole, these NK patients could not be separated from this section. Although Diaz-Valle et al., Balal et al., and Serrano-Gimenez et al. did not utilize control groups, their patients all had been refractory to standard treatments, and most patients subsequently experienced complete re-epithelialization within one to two months after starting topical insulin [32,45,47]. Four patients in Diaz-Valle et al.’s case series did not achieve complete resolution, but they still achieved a mean reduction of 91.5% in their corneal defects. Additionally, Balal et al. noted that no recurrence of PEDs was noted in their study group [32]. Diaz-Valle et al. and Esmail’s controlled studies comparing topical insulin to autologous serum or artificial tears demonstrated that insulin use provided a faster rate of wound healing and smaller defect size at evaluated time points [31,46]. Despite these findings, further studies need to be conducted to evaluate the differences in the efficacy of topical insulin for the various etiologies described in these studies.

## Conclusions

The reviewed literature underscores the promising role of topical insulin in promoting corneal epithelial healing across a spectrum of corneal pathologies. For DK, topical insulin emerged as a superior option compared to conventional therapy, accelerating wound closure and potentially restoring limbal stem cell function. The findings suggest that a lower concentration of 0.5 IU/mL may optimize healing outcomes in DK-associated PEDs. However, for DED, the evidence remains inconclusive, with conflicting results regarding the efficacy of topical insulin. While some studies report improvements in PROs and corneal staining, others found no additional benefits compared to standard artificial tears. Further research is needed to elucidate the role of topical insulin in non-diabetic DED.

Moreover, for NK and PEDs of various etiologies, topical insulin showed promising results in promoting epithelialization and restoring corneal sensation, particularly in cases refractory to standard treatments. Animal studies support the notion that topical insulin may facilitate nerve regeneration and corneal wound healing through the activation of cellular signaling pathways. However, controlled clinical trials are warranted to validate these findings and determine the optimal dosing regimens for different corneal pathologies. Overall, the comprehensive review highlights the potential of topical insulin as a novel therapeutic approach in corneal diseases, paving the way for future research and clinical applications.
